# Electrical stimulation: Nonunions

**DOI:** 10.4103/0019-5413.50849

**Published:** 2009

**Authors:** Bauke W Kooistra, Anil Jain, Beate P Hanson

**Affiliations:** Division of Orthopaedic Surgery, Departments of Surgery and Clinical Epidemiology & Biostatistics. McMaster University, 293 Wellington Street North, Suite 110, Hamilton, Ontario, Canada L8L 2X2; 1 Department of Orthopaedics, University College of Medical Sciences, University of Delhi, Delhi - 95, India

**Keywords:** Electric stimulation, fractures, nonunions

## Abstract

The current paper attempts to provide an overview on the currently available fundamental, preclinical, and clinical evidence on the biologic rationale and therapeutic efficacy of electrical stimulation devices applied in patients with long-bone nonunions. Electrical stimulation (ES) involves the generation of an electrical or electromagnetic current through the ununited fracture. Such currents, which are present in physiologically healing bone, provide stimuli that favor a healing response to bone cells. These stimuli include the enhancement of transmembrane and intracellular calcium-mediated signal transduction and an increased synthesis of paracrine and autocrine growth factors by osteoblasts. Favorable healing union rates, ranging from 43% to 90%, as found by several clinical case series, have prompted the orthopedic community to, at least partially, adopt ES for the treatment of long bone nonunions. Nonetheless, randomized controlled trials have not provided definitive evidence of ES causing nonunions to heal more often than sham devices. This impediment is probably formed by small sample sizes, lack of consistency regarding the definition of union and nonunion, and variability in ES current used.

## INTRODUCTION

Nonunion poses a large problem to long bone fractures, occurring in 2–10% of patients,^1^–^4^ as it is associated with high physical morbidity and a heavy psychological burden.^5^ Although defined in variable ways by surgeons in terms of time to radiographic bridging,^6^ nonunions are generally approached by numerous operative interventions, such as internal or external fixation or bone grafts.^7^,^8^ Yet, a more subtle and practical treatment is preferable. On the basis of promising case reports^9^ and laboratory findings,^10^ electrical stimulation (ES) has been introduced to the care of both fresh and nonhealed fractures.

The purpose of this overview is to provide a summary of the available background information and clinical evidence on the use of ES in the treatment of fracture nonunions.

## BIOLOGY OF ELECTRICAL STIMULATION

Musculoskeletal tissues respond to biophysical stimuli, including weight-bearing,^11^,^12^ pulsed ultrasound,^13^ and electrical and electromagnetic fields.^14^ Physiologically, endogenous electrical potentials and currents are generated in these tissues when damaged and terminate when healed.^15^–^17^ These are possibly generated by shear stress piezoelectricity in collagen fibres.^11^ It is thought that these are part of a complex signaling network maintaining normal bone remodeling and fracture healing. That is, these currents may provide a biophysical input to connective tissue cells regarding the mechanical adequacy and needs of the extracellular matrix (ECM).^10^ However, in injured musculoskeletal tissues that are in the early process of healing (called the “lag” phase), electric and electromagnetic currents are still disorganized.^18^,^19^ Possibly, the same is true for nonunions, of which the tissue is, in fact, in a continuous lag phase. In these cases, devices that produce electric or electromagnetic field tend to improve and amplify the physiological healing response by driving electric or electromagnetic currents through damaged tissues.

## DEVICES

Several electric stimulatory devices are employed and have been investigated, varying in the number of coils, the electromagnetic intensity, the pulse frequency, the duration of electric current administration,^20^ and the location of the coils.^7^ Mostly, a device employing a direct electric current (DC), capacitive coupling (CC), or inductive coupling (IC) is used. Introduced by Paterson,^21^ DC involves the generation of an electric current by a cathode and anode, which are placed invasively, adjacent to the bone to be stimulated. Although it allows weight-bearing, two operations are needed to insert and remove the electrodes from the extremity. Conversely, in CC, electric fields are generated by two skin electrodes placed on opposite sides of the bone to be stimulated.^21^ Similar to DC, weight-bearing is possible with CC devices. As for IC, or pulsed electromagnetic field, an external coil mounts an electromagnetic field not only between its cathode and anode, but also in the direction of the nonunion.^7^,^22^,^23^ IC is noninvasive, but does not allow weight-bearing. All devices can be applied in conjunction with any method of stabilization or osteosynthesis. Table 1 summarizes the characteristics of the three devices.

**Table 1:** Characteristics of 3 electrical bone healing stimulation devices

	Invasive	Weight-bearing
Direct current	Yes	Permitted
Capacitive coupling	No	Permitted
Inductive coupling	No	Not permitted

Treatment regimens vary considerably among reports. Some use a fixed treatment period, for example, 12 weeks for DC,^24^ whereas others tailor the treatment duration according to the radiological progression of fracture healing.^21^,^25^ Moreover, the applied electric currents vary across studies in terms of electromagnetic force, pulse frequency, waveform, and duration of emission.^20^

## *IN VITRO* STUDIES

To have connective tissue cells, such as osteoblasts, proliferate and synthesize Extra Cellular Matrix (ECM), they have to be externally stimulated. The mechanisms involved here seem complex, partly in the sense that it comprises the interaction of physical and chemical processes. The osteogenic potential of ES seems to lie in the restoration of the electric field of tissue early in the healing process and amplifying proliferative and other healing–promoting biophysical stimuli in the rest of the healing process.^18^,^19^ Indeed, positive effects of ES on the proliferation and differentiation of osteoblasts have been observed.^26^,^27^ At the cellular level, electric and electromagnetic currents seem to interact with (1) transmembrane signal transduction of cells involved in bone healing, leading to their proliferation, and with (2) the synthesis of growth factors favoring fracture healing, leading to the differentiation and proliferation of osteoprogenitor cells and ECM deposition. These local mechanisms are detrimental, since nonunion cells may be relatively unexposed to endocrine stimuli due to limited vascularity.

The molecular mechanisms of ES induced signal transduction have recently been described by Brighton *et al.*^28^ By using specific metabolic inhibitors, the authors could demonstrate increased DNA production in cultured bone cells following CC stimulation. Yet, when this channel was blocked by verapamil, no such response was noted. For IC, however, the stimulation-induced increase in DNA production was inhibited by inactivation of intracellular calcium stores release. Both the CC and IC scenarios led to increases in intracellular calmodulin. These results suggest that, irrespective of the stimulant device used, a final common pathway exists in the molecular cascade of healing stimulation by ES, that is, an increase in the intracellular calcium, resulting in increased intracellular calmodulin and cell proliferation, respectively.

Apart from the proliferation of cells involved in fracture healing, local growth factors are thought to have an important function in this process. Here, transforming growth factor (TGF) β1, a paracrine protein involved in the proliferation and differentiation of osteochondral cells and in the stimulation of extracellular matrix (ECM) deposition,^29^,^30^ has received considerable attention. Aaron *et al*. have demonstrated increase in healing tissue TGF-β1 levels in response to therapeutic IC fields, but not to placebo stimulation.^31^ Additionally, progenitor cells involved in enchondral bone formation showed upregulated differentiation, ECM synthesis and TGF-β1 expression, while not disorganizing these processes.^32^

## PRECLINICAL STUDIES

Only a few experiments on animals demonstrating the possible efficacy of ES on the healing of nonunions have been conducted. Possibly, this is because nonunion models are hard to establish. Petersson and coworkers have simulated nonunions in rabbit fibulae by placing a silicone spacer in fresh fracture gaps bilaterally.^33^ After 48 days, the spacers were removed, and DC was applied to one fibula and a sham device was applied to the contralateral fibula, serving as a control, for 62 days. Of the six rabbits studied, only one developed overbridging callus on X-ray assessment at the nonunion site, which was stimulated by DC. Conversely, no unstimulated fibulae showed bridging callus. Synostoses were formed both on the stimulated and on the unstimulated side. Histological analysis of the nonunions revealed only slightly more “activity” around the bony ends on the nonunions that were treated with DC.

Kleczynski investigated the effect of DC in a fresh osteotomy model.^34^ He performed fibular osteotomies in 12 rabbits, followed by 21 days of either DC therapy (n=6) or placebo treatment (n=6), in the form of an electrode in situ, which was not connected to the stimulator device. Similar to the study by Petersson and coworkers,^33^ no significant differences in radiographic healing (as measured by the extent of periosteal and endosteal callus) and histological healing reactions (as measured by osteoid formation) were observed. It should be noted, however, that these were qualitative observations.

Despite the inconclusive results of these animal studies on both fresh and ununited fractures and the lack of preclinical evidence of the effects of IC and CC, many studies involving humans have been carried out on this subject. Partly, this is because animal studies could not demonstrate a disadvantage of ES, in the sense that no complications were seen in animal series. Furthermore, the animal studies were small, precluding definite conclusions on the efficacy in animals and, subsequently, in humans.

## CLINICAL STUDIES

### Observational studies

Several exploratory case series have analyzed the outcome of patients with nonunions treated by ES [Table 2]. Although case series have a substantially lower level of evidence (level 4) compared with randomized controlled trials (level 1),^35^ they can sensibly serve to generate hypothesis regarding the examined intervention, not only for fundamental, but also for translational research.^36^ Case series may be more relevant since the wide and subjective inclusion criteria of such studies yield results that are closer to those obtained in routine clinical practice.^37^,^38^ Yet, case series do not entail a comparison or control group, thereby minimizing the strength of inferences on the efficacy of the intervention under study. Moreover, the generalizability of case series findings on the current subject may be inherently small, because ES is used in highly variable ways.^20^

**Table 2:** Results of case series investigating the outcome of nonunions following ES treatment

Study	Sample size	Device	Long bone	Healing rate (%)	Healing rate in infected nonunions (%)
Abeed *et al.*^8^	16	CC	All	69	-
Brighton *et al.*^22^	22	CC	All	77	-
Zamora-Navas *et al.*^42^	22	CC	All	73	100
Ahl *et al.*^45^	23	DC	All	43	-
Brighton *et al.*^44^	178	DC	All	84	74
Kleczynski^37^	34	DC	All	88	-
Bassett *et al.*^24^	24	IC	All	71	-
Bassett *et al.*^46^	46	IC	Tibia	87	79
Bassett *et al.*^47^	83	IC	All	90	86
de Haas *et al.*^48^	17	IC	Tibia	71	-
de Haas *et al.*^26^	44	IC	Tibia	84	82
Dunn *et al.*^25^	31	IC	All	81	-
Heckman *et al.*^49^	149	IC	All	64	60

CC, capacitive coupling; DC, direct current; IC, inductive coupling.

## CAPACITIVE COUPLING

The most recent observational studies on ES have focused on CC, but comprise only small patient groups. The first to describe outcomes of patients with nonunions treated by CC were Brighton *et al.*^21^ The authors treated 22 nonunions for a mean of 25 weeks. Bone union was noted in 17 patients (77%) in a mean of 27 weeks. The only ES-related complication that was noted constituted a local skin rash as a result of the application of an electrode. The authors also reported three cases of osteomyelitis recurring during the ES treatment period, but these are unlikely to have a relation with the ES. After all, CC is noninvasive, not providing an entry for infectious micro-organisms.

Two other, more recent, case series provided healing rates of 69% and 73% following CC treatment during 30 and 26 weeks, respectively.^8^,^3^9 Of note is the 73% union rate found in nonunions with a fracture gap larger than 0.5 cm by Zamora-Navas and coworkers.^39^ Moreover, all eight infected nonunions healed. These acceptable results compared with those of bone grafting for nonunions^40^ has led to comparative studies regarding these two modalities, which will be discussed later in this overview.

## DIRECT CURRENT

Regarding DC, observational evidence on more, though still few, patients is available. Yet, the union rates found by these studies are less consistent than those found in studies focusing on CC. First described by Brighton *et al*., this invasive ES modality was reported not to be associated with any complications, such as infection.^41^ In a relatively large series of 178 nonunions, their 12-week DC regimen, followed by 12 weeks of cast immobilization, was followed by bony union in 84% of all patients and in 74% of patients with a history of osteomyelitis. The authors erroneously attribute the successful healing of their patients to the “correct” application of the DC device, whereas they seek the cause of the remaining nonunions in the “inadequate” electricity applied to those cases. However, as mentioned above, it is impossible to judge whether the successful healings were due to ES or to other factors that were either not measured or not thought of. In consequence, it cannot be deemed validly which type of electrical application is adequate and which is not. Thus, the authors have drawn too aggressive conclusions from their data. Rather, their findings can be used as a motive to conduct more rigorous research.

Similar to the above findings, Kleczynski, in a 34-patient series, found a 88% union rate for mainly tibial nonunions.^34^ Yet the time until healing was remarkable, with a mean of more than 10 months. Ahl and colleagues found a substantially lower proportion (43%) of healed nonunions, and attributed 7 of 13 failures to mobility of the nonunion.^42^

## INDUCTIVE COUPLING

As in laboratory research, IC has received most attention of the three ES devices in clinical case series.^23^–^25^,^43^–^46^ Here, reported consolidation rates range from 64% to 90%. Despite this wide range, these values seem more valid than the union rates reported for CC and DC, because they are based on larger patient samples. In the largest of these series, merely 64% of patients progressed to union.^46^ The authors suggested the restriction of weight bearing, a possibly low adherence, and a lack of experience with and knowledge about IC devices as causes for the relatively low overall healing rate. In another large series, investigated by authors with more experience with IC, union was achieved in 87% of 127 patients.^43^

Although one should be cautious when interpreting subgroup analyses,^47^ patients with osteomyelitis or a history thereof were almost as likely to achieve union as the total study populations [Table 2]. This may indicate that CC is useful for noninvasively treating septic nonunions as opposed to more conventional operative debridements.^4^,^48^

## COMPARING ELECTRICAL STIMULATION AND BONE GRAFTING

Bone grafting is a well-established operative treatment of nonunion. Typically, an autogenous cancellous bone graft is transplanted into the persistent nonunion defect. The graft cells that survive transplantation attract host mesenchymal stem cells by chemotaxis, differentiating into osteoblasts. Ideally, this new, fresh stimulus provides the nonunion tissue with the potential to regenerate. Impressive healing rates of 87–100% in large series have introduced this intervention into practice decades ago.^49^–^52^

Obviously, ES provides advantages over bone grafting, as it is, at least, less invasive. Brighton and *et al*. have compared CC, DC, and bone grafting in a total of 271 patients whose nonunions had been treated by one of these modalities.^53^ In a multivariate analysis, controlling for several prognostic variables such as the type of nonunion (atrophic vs. hypertrophic), the radiologic fracture pattern, and whether the fracture was closed or open, they found no differences in the probability of healing of the nonunion. Specifically, when no such potential risk factors for a nonhealed nonunion were present, the probabilities of a nonunion being healed by 10 months were 99%, 96%, and 99% for CC, DC, and bone grafting, respectively.

Separate comparison of series on ES and series on bone grafting are problematic, because of the small numbers of patients used in ES series. This results in wide confidence intervals surrounding the point estimates of union rates. Moreover, the clinical circumstances and patient populations may differ considerably across different series, thereby diminishing the ability to make fair comparisons between the two interventions.^54^

## RANDOMIZED CONTROLLED TRIALS AND META-ANALYSIS

Unfortunately, despite the growing support of a biologic rationale, only few, small randomized controlled trials have been conducted on the therapeutic efficacy of ES. A recent meta-analysis identified three randomized controlled trials on this topic [Table 3].^20^

**Table 3:** Results of randomized controlled trials investigating the efficacy of ES treatment in nonunions

Study	Sample size	Device	Long bone	Healing rate (%)		Number needed to treat*
					
				Active device	Placebo	
Scott *et al.*58	21	CC	All	55	8	2.2
Barker *et al.* 59	16	IC	Tibia	56	63	-
Simonis *et al.*60	34	IC	Tibia	89	50	2.6

* According to the difference in union rates between ES- and placebo-treated patients

CC, capacitive coupling; DC, direct current; IC, inductive coupling.

Scott and King randomized 23 patients with treated long-bone nonunions to either a CC or a sham device.^55^ They defined a nonunion as the presence of a fracture for 9 months or longer and no clinical or radiological signs of progress for the most recent 3 months. Only patients who had not been treated otherwise than immobilization in a cast for the most recent 3 months were included. Additionally, patients received no other additional therapy to promote healing. Six months after the initiation of the intervention, 6 of 11 nonunions treated with an active device had healed, as opposed to only one of 12 cases treated with a sham device (*P* = 0.02). Yet, when calculated as a relative risk, this association is not significant.^20^ Thus, these results have provided preliminary evidence that ES is efficacious for treating nonunions, but larger sample sizes and similar results in other randomized controlled trials would be needed for more convincing evidence.

Yet, the results of another randomized controlled trial are contradictory and based on an even smaller sample of patients,^56^ when compared with the trial described above. Here, only patients with tibial nonunions were included. Furthermore, nonunion was defined as the presence of a fracture line on radiographs and of clinical mobility for at least a year and not improving for the most recent 3 months. The device was used for 24 weeks. Similar to the trial by Scott and King,^55^ no treatment other than the device and a cast was given. This standardization of care strengthens the methodology of the study, since unions or nonunions will not have been caused by treatment other than ES, as could have been the case in another trial.^57^ No difference in union rates at 24 weeks was found between the active and dummy device groups; five of the nine patients allocated to the active device healed, when compared with five of the eight controls. Similarly, no difference was noted in pain and tenderness at the fracture site at the same time point. The authors go on to conclude that IC may not have an adjunctive value in the treatment of tibial nonunions over that of conventional treatment. Further, they state that healing responses attributed to ES may, in fact, be due to the conservative casting regimen itself. Yet, as the methods section of the report states that for dummy devices the electrical current was diverted to an internal load inside the device, there are concerns that controls were exposed to weak electromagnetic fields as well.^20^ Thus, the effect of IC stimulation may be partially masked by comparing the active and control groups.

More recently, Simonis and colleagues performed a similar trial on tibial nonunions existing for at least 1 year, showing no progression in the 3 most recent months.^57^ Patients were treated with an IC or sham device for 6 months and received a unilateral external fixator with compression. Patients in the active treatment group had progressed to union significantly more often than had controls (16/18 vs. 8/16, respectively, *P* = 0.02). However, there were more smokers in the control group, possibly favoring active ES.^58^ Indeed, when adjusted for this difference in smoking status, no significant difference of IC stimulation could be detected any more (*P* = 0.07). Therefore, the authors' conclusion that “electric stimulation is an effective adjunct to the conventional treatment of tibial nonunions” should be assessed with care.

## Limitations of Randomized Controlled Trials on Electric Stimulation

Several limitations to the described randomized controlled trials exist. First, both union and nonunion were defined in variable ways. This is common to orthopedic practice,^6^,^59^ but it complicates the interpretation of the results of these trials all the same. The less conservative definition of nonunion (i.e., a fracture being present for at least 9 months) used by Scott and King^55^ may have led to the inclusion of patients with less recalcitrant nonunions than those in the other two trials.^56^,^57^ Furthermore, union was defined differently across the studies, one only using radiographic^57^ and two using a combination of radiographic and clinical criteria.^55^,^56^ Moreover, the employed radiographic criteria were dissimilar for all three studies.

In addition, both CC and IC devices were investigated, which limits the comparability of the trials because of different electrical properties. Moreover, even between the IC trails, the frequencies and the durations of the pulsed fields differed (15 vs. 23 Hz and 5 vs. 3 ms, respectively).^56^,^57^ Also, Scott and King investigated nonunions of all long bones, while the other two trials solely focused on tibial fractures.

Possibly, these factors can explain the substantial statistical heterogeneity as found by a recent meta-analysis (I^2^ = 60%).^20^ When pooling the results of the abovementioned randomized controlled trials with another trial regarding delayed union, no significant advantage of ES over placebo was found in terms of union, but there was a trend in favor of ES (relative risk of union = 1.76, 95% confidence interval = 0.81–3.80). The authors of the meta-analysis state that the wide use of ES in the orthopedic community is not justified by the current best available evidence.^20^ Indeed, and in addition to the limitations mentioned above, the small total sample of randomized patients obviates a just conclusion to be drawn about the therapeutic efficacy of ES. Therefore, “neither enthusiastic dissemination nor confident rejection of this therapeutic modality is justified”.^20^

## DISCUSSION

ES represents a 500 billion dollar market in the United States.^20^ It is therefore worthwhile to base appliance of ES for nonunion patients on sound scientific evidence

The current frequent use of ES devices in the treatment of nonunions^60^ is supported by a growing body of fundamental, laboratory findings,^10^ but seems not to be justified on the basis of sound, pragmatic evidence.^20^ The presently found discrepancy between the initial enthusiasms surrounding ES because of favorable results found in several case series and the modesty following pooling of high-quality comparative evidence underlines the need for a more conservative state of mind as to the prescription of ES devices. This is illustrated by the fact that even before the publication of the first randomized controlled on the effect of IC, more than 11,000 devices had been used worldwide for nonunions.^61^ Similarly, the true efficacy of DC has never been investigated in a randomized and blinded comparison, leaving its treatment effect subject to speculation.^38^

Acquiring a comprehensive image of the impact of the currently best available evidence on clinical practice is complicated by the variability in the definition of nonunion. Some orthopedic surgeons define it only clinically, whereas others solely use radiographic, and some others use both.^6^ Consequently, not only do researchers include different patients; orthopedic surgeons may also apply these results erroneously to their own patients. After all, on average, orthopedic surgeons define nonunion as the absence of a healing response for 6.3 months.^6^ Yet, the results of randomized controlled trials only apply to patients without signs of healing for 9 or 12 months.

In conclusion, although preclinical and observational evidence seems to provide a sensible rationale for using ES in the treatment of long bone nonunions, the current paucity of and heterogeneity in sound clinical evidence prevent orthopedic surgeons from justifiably implementing it.
